# Effect of corticosteroids on mortality in patients with community-acquired pneumonia

**DOI:** 10.1186/s13054-023-04645-w

**Published:** 2023-09-19

**Authors:** Shangzhong Chen, Caibao Hu

**Affiliations:** Department of Intensiventensive Care, Zhejiang Hospital, 1229 Gudun Road, Hangzhou, 310013 Zhejiang China

To the Editor,

We read with great interest the recent meta-analysis by Dr. Wu et al. [1], which investigated the efficacy of corticosteroids in patients with severe community-acquired pneumonia (CAP). A total of severe randomized controlled trials involving 1689 patients were included, and the primary finding is that in patients with severe CAP, adjunctive corticosteroids can provide survival benefits and improve clinical outcomes without increasing adverse events. We have some different opinions.

First, in the sensitivity analysis, this review used leave-one-out analyses (removing one trial at a time from each meta-analysis), and they reported that all the results remained stable. However, this result may be biased by inadequate study inclusion. We performed another literature search on the basis of the previous systematic reviews and identified another two trials (the Santeon-CAP Trial [2] and Snijders et al.’s Trial [3]) that should be included. After the inclusion of these two trials, the sensitivity analysis became unstable in the leave-one-out analysis, and the result became non-significant after excluding Dequin et al.’s trial [4] (Fig. 1, overall effect: RR 0.77, 95% 0.57–1.03). Besides, two recent adequately powered, rigorously designed randomized trials [4, 5] of severe CAP have reported opposite outcomes, suggesting that the efficacy of corticosteroids in severe CAP may be heterogeneous. In addition, the instability of the results of the current meta-analysis suggests that further studies may be needed.

**Fig. 1:**
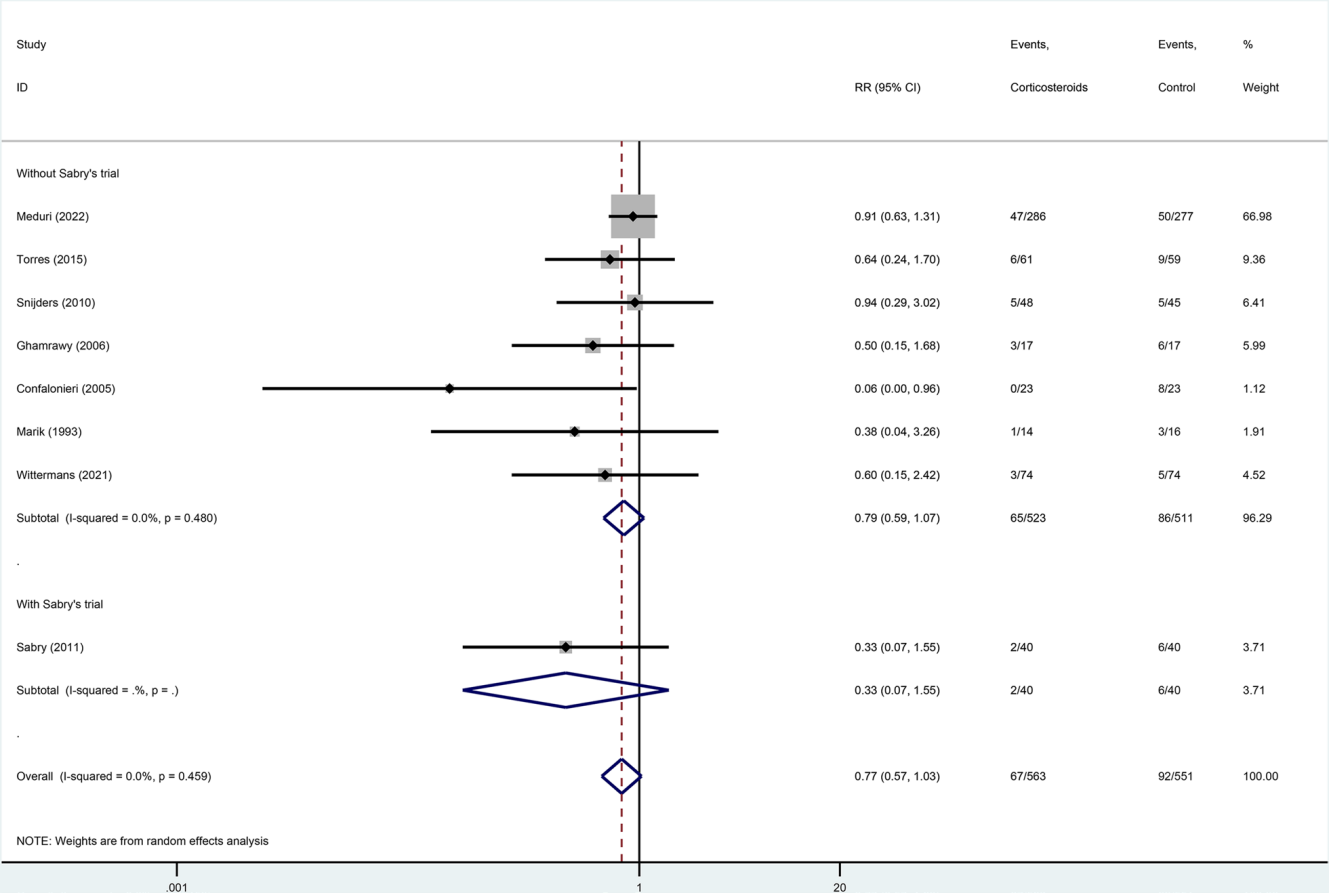
sensitivity analysis of the effect of corticosteroids on mortality in community-acquired pneumonia. *Note*: in the leave-one-out sensitivity analysis, the effect on all-cause mortality became non-significant after excluding Dequin et al.’s trial

Second, within all included trials, there is some concern [6] about Sabry’s [7] trial. All included trials reported in-hospital or 30-day mortality as the outcome. However, in Sabry’s trial, the authors used extreme short-term mortality (8-day) as the primary outcome, which was less than the reported median time to death in CAP (nine days, which means almost half of all deaths were not observed) [8]. Thus, to reach a stable result, it is reasonable to perform another sensitivity analysis by excluding this trial. In Fig. 1, both pooled analysis with or without Sabry’s [7] trial was performed and the result remained non-significant.

## Data Availability

Not applicable.
